# The *open for business* model of the bithorax complex in *Drosophila*

**DOI:** 10.1007/s00412-015-0522-0

**Published:** 2015-06-12

**Authors:** Robert K. Maeda, François Karch

**Affiliations:** Department of Genetics and Evolution, University of Geneva, 30 quai E. Ansermet, 1211 Geneva-4, Switzerland

**Keywords:** Chromatin domains, Boundaries, Insulators, Bithorax complex

## Abstract

After nearly 30 years of effort, Ed Lewis published his 1978 landmark paper in which he described the analysis of a series of mutations that affect the identity of the segments that form along the anterior-posterior (AP) axis of the fly (Lewis 1978). The mutations behaved in a non-canonical fashion in complementation tests, forming what Ed Lewis called a “pseudo-allelic” series. Because of this, he never thought that the mutations represented segment-specific genes. As all of these mutations were grouped to a particular area of the *Drosophila* third chromosome, the locus became known of as the bithorax complex (BX-C). One of the key findings of Lewis’ article was that it revealed for the first time, to a wide scientific audience, that there was a remarkable correlation between the order of the segment-specific mutations along the chromosome and the order of the segments they affected along the AP axis. In Ed Lewis’ eyes, the mutants he discovered affected “segment-specific functions” that were sequentially activated along the chromosome as one moves from anterior to posterior along the body axis (the colinearity concept now cited in elementary biology textbooks). The nature of the “segment-specific functions” started to become clear when the BX-C was cloned through the pioneering chromosomal walk initiated in the mid 1980s by the Hogness and Bender laboratories (Bender et al. 1983a; Karch et al. 1985). Through this molecular biology effort, and along with genetic characterizations performed by Gines Morata’s group in Madrid (Sanchez-Herrero et al. 1985) and Robert Whittle’s in Sussex (Tiong et al. 1985), it soon became clear that the whole BX-C encoded only three protein-coding genes (*Ubx*, *abd-A*, and *Abd-B*). Later, immunostaining against the *Ubx* protein hinted that the segment-specific functions could, in fact, be *cis*-regulatory elements regulating the expression of the three protein-coding genes. In 1987, Peifer, Karch, and Bender proposed a comprehensive model of the functioning of the BX-C, in which the “segment-specific functions” appear as segment-specific enhancers regulating, *Ubx*, *abd-A*, or *Abd-*B (Peifer et al. 1987). Key to their model was that the segmental address of these enhancers was not an inherent ability of the enhancers themselves, but was determined by the chromosomal location in which they lay. In their view, the sequential activation of the segment-specific functions resulted from the sequential opening of chromatin domains along the chromosome as one moves from anterior to posterior. This model soon became known of as the *open for business* model. While the *open for business* model is quite easy to visualize at a conceptual level, molecular evidence to validate this model has been missing for almost 30 years. The recent publication describing the outstanding, joint effort from the Bender and Kingston laboratories now provides the missing proof to support this model (Bowman et al. 2014). The purpose of this article is to review the *open for business* model and take the reader through the genetic arguments that led to its elaboration.

## Introduction

### A quick overview of the model of Ed Lewis

**Fig. 1:**
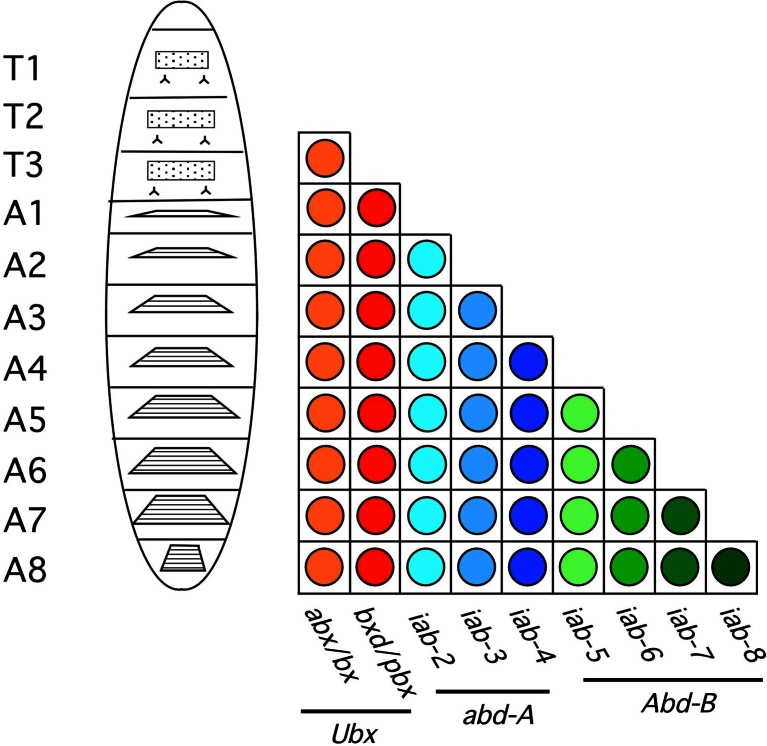
The model of Ed Lewis. Reproduced from Fig. 2 of Maeda and Karch (2006) with the permission of the Company of Biologists; DOI: 10.1242/dev.02323. A larva is drawn on the left with its three thoracic segments marked as T1, T2 and T3 as well as its eight abdominal segments marked A1 to A8. The diagram next to the larva represents the presence of absence of a segment-specific function that is required for the specification of each segment. As the segments-specific functions are aligned on the chromosome (in the x-axis) in the same order as the segments along the body axis, the diagram is represented in the form of a matrix. The fact that mutations in a given segment-specific function always transform that segment into the copy of the immediately adjacent segment anteriorly implies that the more anterior segments-specific functions are active in that segment. This led Ed Lewis to propose that segment-specific functions act in an additive fashion. The *Ubx*, *abd-A*, and *Abd-B* genes are indicated below the segment-specific functions, with the horizontal lines defining the mutations that are not complemented by the respective *Ubx*, *abd-A*, or *Abd-B* mutations

*Drosophila* embryos and larvae harbor a head, three thoracic segments (T1–T3) and eight abdominal segments (A1–A8; see left panel of Fig. 1). At metamorphosis, the eighth abdominal segment gives rise to parts of the genital structures of the adult fly. When the whole BX-C is deleted, mutant embryos die before hatching, but at a stage where it is already possible to recognize the identities of the segments. Thus, it is possible to see that mutants lacking the BX-C have all posterior segments from T3 transformed into copies of T2 (to be precise, these transformations affect parasegments—see below—but Lewis talked in terms of segments). This finding led Ed Lewis to consider T2 as the ground state of development on which the activity of the BX-C built, thereby assigning identities to the more posterior segments (Lewis 1978) (Fig. 1).

There are other mutations within the BX-C that primarily affect the identity of single segment under the control of the BX-C. Many of them allow survival to adulthood. These mutations define the nine “segment-specific functions”, *abx/bx*, *bxd/pbx*, *and iab-2 through iab-8* that specify the identities of T3 and all eight abdominal segments (A1 through A8), respectively. Typically, loss-of-function mutations in the BX-C result in the transformation of a given segment into a copy of the segment directly anterior to it. The fact that mutations in individual “segment-specific functions” always cause transformations toward the segment immediately anterior to them and not toward the ground state (T2) indicates that everything required for the identity of the more anterior segments still functions in the more posterior segments. Thus, Ed Lewis proposed that the segment-specific functions act in an additive fashion: once they are turned on in the segment they specify, they remain active in the more posterior segments. Lewis synthesized these findings into two rules for BX-C regulation: “… a [segment-specific function] derepressed in one segment is derepressed in all segments posterior thereto…” and “the more posterior a segment… the greater the number of BX-C [segment-specific functions] that are in a derepressed state” (Lewis 1978). These rules are illustrated in the form of a matrix in which the anterior-posterior axis of the fly is represented along the *y*-axis and the activity state of the BX-C is represented along the *x*-axis (see Fig. 1).

### The segment-specific functions are segment-specific enhancers

Three classes of mutations (*Ubx*, *abd-A*, and *Abd-*B) associated with embryonic lethality also exist within the BX-C. They cause the transformation of a group of segments into a more anterior segment (Lewis 1978; Sanchez-Herrero et al. 1985; Tiong et al. 1985). For example, *Ubx* (*Ultrabithorax*) mutant embryos have their T3 and A1 segments transformed into T2, as if both the *abx/bx* and *bxd/pbx* segment-specific functions were inactivated in *Ubx* alleles. In agreement with Ed Lewis’ observations, *Ubx* mutations fail to complement *abx*, *bx*, *bxd*, or *pbx* alleles. This lack of complementation is contrasted by the observation that heterozygous flies, with *bx* or *abx* mutations on one chromosome and *bxd* or *pbx* mutations on the other, looked normal. Ed Lewis proposed the term “pseudo-allelism” to describe these conflicting observations.

**Fig. 2:**
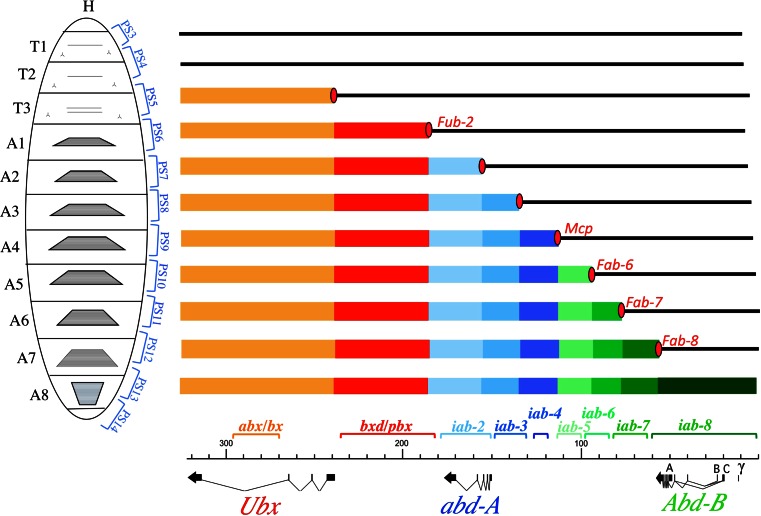
The *open for business model*. A larvae is represented on the left with the thoracic (T1–T3) and abdominal segmental boundaries (A1–A8) as well as the corresponding parasegmental boundaries (PS1–PS14; see text). The genomic map of the BX-C is drawn on the *x*-axis at the scale indicated in kilobases. The *Ubx*, *abd-A*, and *Abd-B* transcription units are drawn at scale below the genomic map. The extent occupied by the segments-specific functions are indicated by brackets above the DNA line. The sequential opening of the segment-specific regulatory domains is drawn for each parasegments. While *colored rectangles* indicate *open for business*, the *solid black line* represents closed chromatin (see text). Boundaries marking the borders between the open and closed domains are shown by *red ovals*. The boundaries that have been identified by mutational analysis are named. Note the similarity with the model of Ed Lewis where the *dots* shown in Fig. 1 are replaced by DNA domains

After the discovery that the BX-C encodes only three genes (*Ubx*, *abd-A*, and *Abd-B*), the phenomenon of pseudo-allelism was finally explained. In situ hybridization and antibodies generated against these proteins allowed the determination of their expression patterns (Akam 1983; Beachy et al. 1985; Bender et al. 1983a; Casanova et al. 1987; Celniker et al. 1990; Karch et al. 1990; Karch et al. 1985; Macias et al. 1990; Sanchez-Herrero 1991; White and Wilcox 1985). By staining various mutant embryos, it was finally understood that the “segment-specific functions” corresponded to *cis*-regulatory regions that regulate the expression of *Ubx*, *abd-A*, or *Abd-B* in a segment-specific fashion. The molecular organization of the BX-C is shown along the *x*-axis of Fig. 2, with the extent of each of the nine segment-specific function indicated by brackets above the DNA line. The *Ubx*, *abd-A*, and *Abd-B* transcription units are shown below. The regulatory interactions between the segment-specific functions and their respective target promoter follow a color code (Figs. 1 and 2). While the *abx/bx* and *bxd/pbx* regions regulate *Ubx* (as indicated in reddish color), *iab-2* though *iab-4* regulate *abd-A* (blueish). Finally, *iab-5* through *iab-8* regulate *Abd-B* (greenish; see (Maeda and Karch 2006) for review).

It should be noted that the embryonic expression patterns of *Ubx*, *abd-A*, and *Abd-B* (and some other homeotic genes) are made up of reiterated units along the AP axis. Each unit of expression is roughly equivalent to one segment in length, but slightly shifted relative to the morphological body segments that appear during mid embryogenesis. These units are known as parasegments (Martinez-Arias and Lawrence 1985). One parasegment (PS) is composed of the posterior compartment of one segment and the anterior compartment of the next segment. For example, PS5 corresponds to the posterior part of T2 and the anterior part of T3. For the most part, the parasegment-segment shift is rarely mentioned due to the fact that the visible adult cuticle is mostly made up of cells from the anterior half of each segment. Nevertheless, the correspondence between parasegments and segments are indicated in the figures of this paper.

#### Regulatory domains; the ***open for business*** model

The finding that the segment-specific functions are in fact *cis*-regulatory elements clarified the genetic schema that Ed Lewis had been working on for decades. Due to the size of the regulatory regions in question (from 10 to 60 kb), multiple enhancers were hypothesized to exist within each regulatory domain. This was supported by some of the early work using *bxd* mutations. There are many *bxd* mutations caused by chromosome breaks. These mutations make up an allelic series with differing strengths of transformation (lowering the *Ubx* expression in PS6). It turns out that mutations with breakpoints closer to the *Ubx* promoter cause stronger transformations, while mutations with breakpoints further away from the promoter cause weaker transformations (Bender et al. 1983b; Bender et al. 1985). The correlation between the loss of *Ubx* expression in PS6 and the amount of DNA from the *bxd/pbx* region that was separated from the *Ubx* target promoter [finally published 26 years later in Pease et al. (2013)] was taken as evidence for the existence of multiple enhancers.

Enhancers function from a variety of positions with respect to their target promoters and can often activate different promoters, depending on the circumstances. Given this promiscuity, clustering of the BX-C enhancers in discrete regions along the chromosome was puzzling. Peifer et al (1987) brought a plausible explanation to this question with the idea that parasegmental/segmental address may be conferred by the DNA domain in which the enhancers reside (Fig. 2). According to their view, each regulatory region should be a chromosomal domain that opens up in the appropriate parasegments during early embryogenesis, enabling the enhancers residing within the domain to perform their regulatory “business” with the target promoter (dubbed as the *open for business* model in Akam et al. 1988).

The idea that BX-C enhancers might be regulated coordinately through chromatin domains primarily came from the analysis of dominant gain-of-function (GOF) mutations, where a given segment develops like a copy of the segment that lies immediately posterior to it. Peifer et al (1987) focused on the dominant *Cbx*^*1*^ mutation to generate their model, but later work describing mutations that delete boundary elements separating regulatory domains also supported this idea. Two additional lines of evidence also pointed to coordination of enhancers within a chromatin domain. The first was the recovery of enhancer trap transposons within the BX-C that brought forward a visual argument to the segment-specific regulatory domain model. And secondly, experiments where special, early enhancers (initiators) were exchanged between different domains, were able to consolidate the model by entirely fulfilling the predictions made by the open for business model.

#### The ***Cbx***^*1*^ mutation

**Fig. 3:**
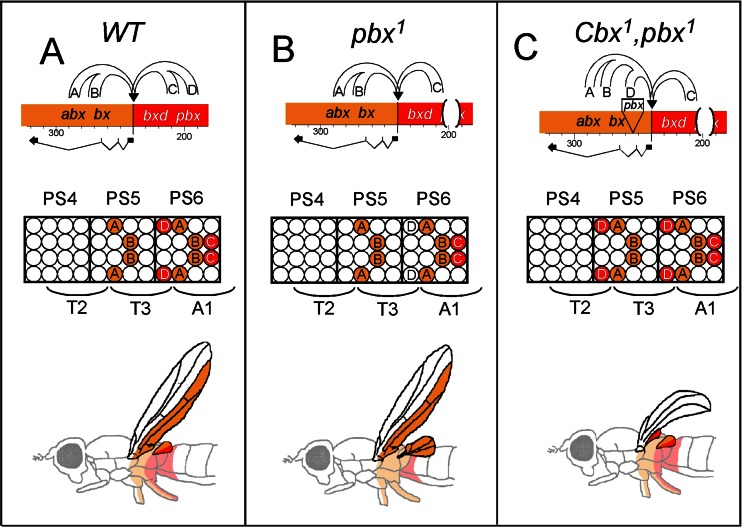
Molecular genetics of* Cbx* ^*1*^. Modified from Fig. 4 of Peifer et al. 1987 and from Fig. 1.5 of Maeda and Karch (2009); DOI: 10.1016/S0070-2153(09)88001-0 with the permission of CSH Press and Elsevier, respectively. The *abx/bx* (*orange*) and *bxd/pbx* (*red*) regulatory regions, respectively active in PS5 and PS6 are represented on the top of each of the **a**, **b**, and **c** panels. A cartoon of the central nervous system in PS4, PS5, and PS6 is represented in the middle of each panel with the parasegmental borders on top and the corresponding segmental borders below. At the bottom of each panel, an adult thorax is shown with the PS5- and PS6-specific expression of *Ubx* drawn in *orange* and *red*, respectively. Note the PS5-PS6 boundary passing through the middle of the haltere. In panel **a**, enhancers A and B from the *abx/bx* regulatory domain turn on *Ubx* at a moderate level into the A and B cells of the CNS. These A and B enhancers remain active in the more posterior parasegments. Note that the C and D enhancers of the *bxd/pbx* regulatory region remain inactive in PS5. In PS6 however, these C and D enhancers activate *Ubx* at a higher level in the C and D cells. Panel **b** displays the *pbx* ^*1*^ mutation deleting the D enhancer. As a consequence, the D cells located in the posterior part of T3 do not express *Ubx*, leading in adults to the transformation of the posterior part of the haltere into posterior wing. In the *Cbx* ^*1*^ mutation (panel **c**), the D enhancer is relocated within the *abx/bx* regulatory, enabling them to function in PS5, as drawn in the cartoon of the CNS. This activity leads to the transformation of the posterior part of the wing into the posterior part of the haltere

*Cbx*^*1*^ is a gain-of function mutation that transforms the posterior half of the wing (T2) into the posterior half of the haltere (T3). Fine structure mapping led Lewis to discover that the original *Cbx*^*1*^ chromosome contained two, separable mutations. One of them was associated with the dominant GOF phenotype and the other one was associated with a recessive phenotype. The recessive mutation was named *postbithorax*^*1*^ (*pbx*^*1*^) in accordance with the transformation of the posterior haltere into the posterior wing (Lewis 1954). Based on this phenotype, Lewis reasoned that the *pbx*^*+*^ function must be to “make” the posterior haltere. Given that the dominant GOF *Cbx*^*1*^ phenotype is to transform the posterior wing into the posterior haltere, it followed that *Cbx*^*1*^ must cause the expression of the *pbx*^*+*^ function one segment ahead, in T2. In 1983, the molecular lesions associated with the *Cbx*^*1*^ mutation were identified by Welcome Bender and confirmed Ed Lewis’ genetic predictions (Bender et al. 1983a). Bender found that in *Cbx*^*1*^, a 17-kb piece of DNA had been excised from the *bxd/pbx* region and transposed in reverse orientation 40 kb away within the second intron of *Ubx* (Fig. 3c). The deletion alone (*pbx*^*1*^) abolishes *Ubx* expression in the posterior half of the haltere imaginal disc (Fig. 3b), but its relocation 40 kb upstream activates *Ubx* expression in the posterior part of the wing imaginal disc (Cabrera et al. 1985; White and Akam 1985; White and Wilcox 1985). The loss of *Ubx* expression in the posterior compartment of the haltere disc in *pbx*^*1*^ mutants indicated that the 17-kb-long DNA element deleted enhancers responsible for *Ubx* expression in these cells (Fig. 3b). Given the positional flexibility of most enhancers, if these enhancers autonomously controlled their activity along the AP axis, then moving them from their endogenous location to the second intron of *Ubx* would not be expected to affect their function. And yet, moving these enhancers 40 kb changed the parasegment in which they activate *Ubx*. These observations suggested that position along the chromosome determines where BX-C enhancers are active along the AP axis. As the BX-C was first defined by segment-specific functions, a likely extension of the *Cbx*^*1*^ result would be that each segment-specific function derived from a region of the chromosome where enhancers were coordinately regulated along the AP axis. A model summarizing this idea is shown in Fig. 3 where a number of cell-type-specific enhancers from the *abx/bx* and *bxd/pbx* are depicted (A, B, C and D). In PS5, the *abx/bx* DNA domain opens up, enabling the A and B enhancers to activate *Ubx* in the A and B cells of PS5. As the domain remains open in more posterior parasegments (first rule of Ed Lewis model, see above), the A and B enhancers remain active as well in those more posterior parasegments (Fig. 3a). In the meantime, the *bxd/pbx* domain remains inactive in PS5 (see also Fig. 2). In PS6, the next adjacent domain (*bxd/pbx*) opens up, enabling the C and D enhancers to activate *Ubx* in different cell types (Fig. 3a). In *Cbx*^*1*^, the D enhancers are relocated in the domain that is active in PS5, enabling them to activate *Ubx* one parasegment ahead of their normal realm of activity (Fig. 3c). In this view, BX-C enhancers provide cell-type or tissue specificity and their location along the chromosome provides the segment/parasegmental information about where the enhancers should be activated along the AP axis.

#### The ***Mcp*** and ***Fab-7*** boundary deletions

At the time of the proposal of the open for business model, *Mcp*^*1*^ (isolated by Lynn Crosby in Ed Lewis’ laboratory) was another GOF mutation that had been localized on the DNA map (Karch et al. 1985). For classical geneticists, dominant GOF mutations are enticing treats. To gain more insights into the mechanisms underlying a dominant mutation, the geneticist simply follows the tried-and-true method of inducing second site mutations that revert the dominant phenotype. For instance, Ed Lewis performed many screens to revert the *Cbx1* phenotype. Nearly all revertants turned out to be chromosomal rearrangement breaks within the 70-kb-long *Ubx* transcription unit. This observation suggested that *Cbx*^*1*^ was causing misexpression of *Ubx*.

The *Mcp*^*1*^ mutation turned out to be a 3 kb deletion located near the region defined by mutational analysis as *iab-5* (Fig. 2). However, while *iab-5* mutations lead to an A5 to A4 transformation, *Mcp*^*1*^ causes the dominant transformation of A4 into A5. It was, therefore, thought that the deletion caused misexpression of *iab-5* in A4, perhaps by removing a repressor involved in *iab-5* repression in segments anterior to A5. The finding of *Mcp* revertants with rearrangement breakpoints in *iab-5* confirmed the assumption that the deletion affected *iab-5* regulation (Karch et al. 1985).

In the light of the *open for business* model, the location of *Mcp*^*1*^ at the border between the *iab-4* and *iab-5* regulatory domains inspired another interpretation. If the chromosomal domains were important for coordinately regulating BX-C enhancers in a parasegmentally controlled manner, then there must be some mechanism to limit the area of one domain from the area of the next. This interpretation would predict the presence of domain boundary elements. Accordingly, the *Mcp*^*1*^ deletion was thought to possibly be the deletion of a boundary element separating the *iab-4* and *iab-5* regulatory domains. In the context of the *Mcp*^*1*^ mutation, opening of the *iab-4* domain in A4 would spread to *iab-5*, leading to the ectopic activation of *iab-5* enhancers in A4.

**Fig. 4:**
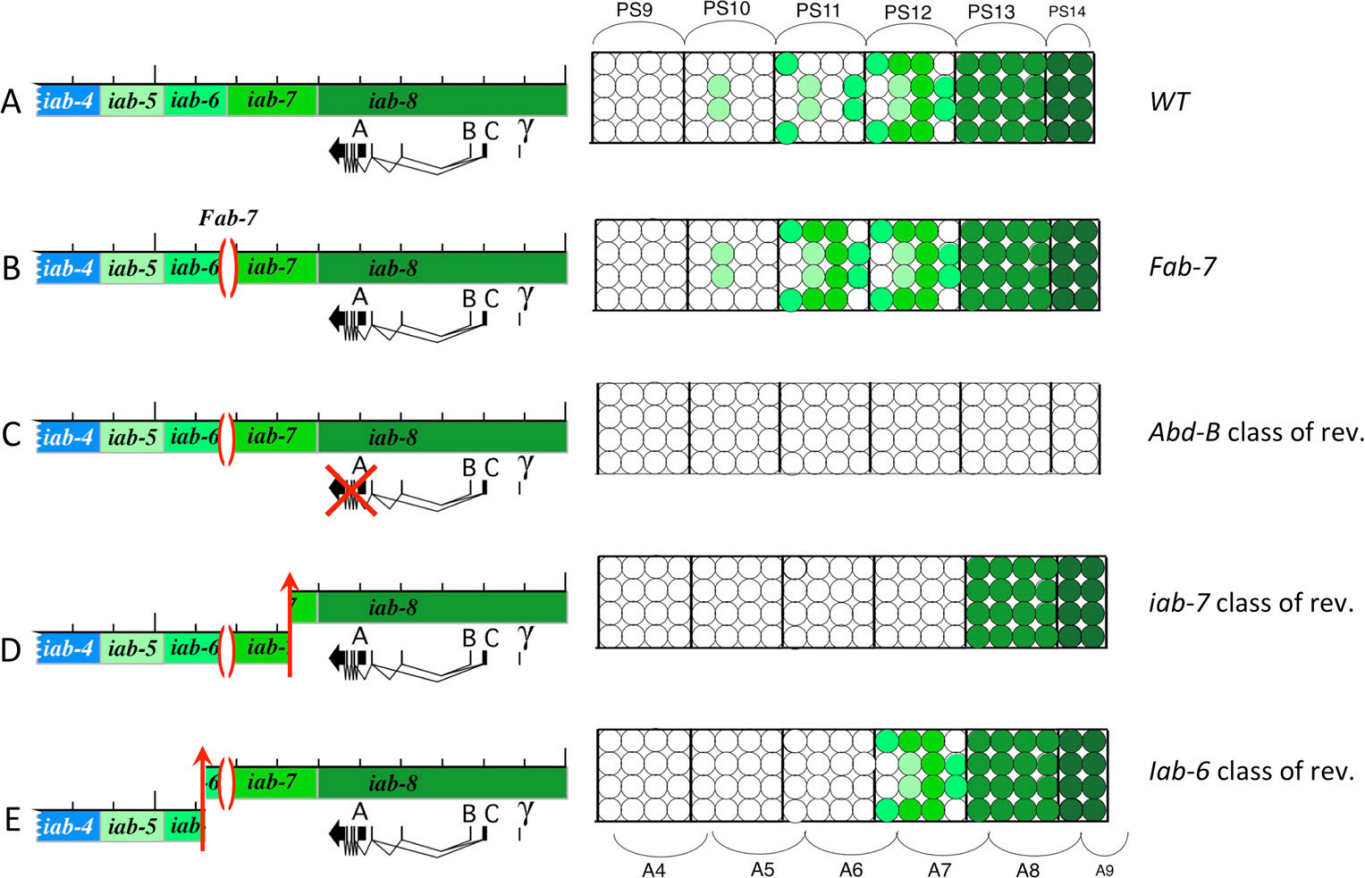
*Fab-7* mutation and revertants. The *Abd-B* transcription unit and associated regulatory domains *iab-5* through *iab-8* are drawn on the left of each panel. The parasegment-specific expression pattern of *Abd-B* are represented on the right in the form of cartoon of the central nervous system (with parasegmental and segmental borders indicated respectively above and below). In WT, panel **a**, *Abd-B* is expressed at a low level in a few cells in PS10. This expression is controlled by the *iab-5* regulatory domain. In PS11, a few more cells express *Abd-B* at a slightly higher level, under the control of *iab-6*. In PS12 additional cells express *Abd-B* at a higher level, under the control of the *iab-7* domain. Finally, in PS13, *Abd-B* appears in all cells at a higher level, under the control of *iab-8*. It should be noticed that in PS14, a truncated form of *Abd-B* is expressed from alternate promoters (B, C, and γ) at even a higher level. The enhancers controlling *Abd-B* expression in PS14 are not known. In panel **b**, the *Fab-7* ^*1*^deletion located between *iab-6* and *iab-7* is drawn on the genomic map. This deletion leads to the ectopic activation of *iab-7* in PS11, resulting in the appearance of the PS12-specific *Abd-B* expression pattern in PS11. Panel **c** represents the first class of *Fab-7* ^*1*^ revertants that inactivate *Abd-B*. This class confirmed that *Fab-7* ^*1*^ affects *Abd-B* regulation. Panel **d** represents the second class of *Fab-7* ^*1*^ mutations that map within the *iab-7* region. As the rearrangement breakpoints separate *iab-5*, *iab-6*, and part of *iab-7* from their *Abd-B* target promoter, *Abd-B* expression is lost in PS10, PS11, and PS12. This class of revertants confirmed that *Fab-7* ^*1*^ is misregulating *iab-7*. Finally, the 3rd class of *Fab-7* ^*1*^ revertants in which a rearrangement breakpoint occurred in *iab-6* is represented in panel **e**. Overall, this analysis established that the *Fab-7* ^*1*^ GOF phenotype appears only if the whole region from *iab-6* to the *Abd-B* transcription is intact in *cis*

In 1985, the discovery of another GOF mutant, *Fab-7*^*1*^ by Henrik Gyurkovics in Szeged, brought additional support to the concept of boundaries delimiting BX-C regulatory domains (Gyurkovics et al. 1990). In the case of the *Fab-7* mutation, a 4.3-kb-long deletion occurred in the region delimiting *iab-6* from *iab-7* (Fig. 4b) and caused an A6 to A7 transformation. Following Ed Lewis’ model, the *iab-7* function seems to be activated ectopically in A6. As for *Mcp*^*1*^, the simplest interpretation of *Fab-7* consists in assuming that the deletion removes the binding site of a repressor/silencer complex that normally keeps *iab-7* inactive in segments anterior to A7. But again the isolation and localization of revertants of *Fab-7*^*1*^ make this simple interpretation unlikely. In this reversion screen, *Fab-7*^*1*^ homozygotes were mutagenized with X-rays and crossed to WT females. The progeny of this cross would be expected to be heterozygous for the *Fab-7*^*1*^ mutation and show the dominant transformation of A6 into A7 unless the X-ray treatment hit a region necessary for the manifestation of the GOF phenotype. Figure 4 summarizes the three classes of revertants that were recovered during this simple screen (Gyurkovics et al. 1990). The first class corresponded to *Abd-B* alleles (Fig. 4c). These chromosomes do not produce any *Abd-B* protein, confirming thereby that the *Fab-7*^*1*^ mutation affects *Abd-B* regulation. The second class of revertants carry chromosomal rearrangements breakpoints within the *iab-7* domain (Fig. 4d). In these mutants, the *Fab-7*^*1*^ deletion along with *iab-6* and *iab-5* are separated away from the *Abd-B* target gene, causing the loss of *Abd-B* expression in A5/PS10 to A7/PS12. Homozygotes for such revertants are viable and have their A5/PS10 through A7/PS12 that develop like a copy of A4/PS9. This class of revertants confirms that *iab-7* must be intact and in *cis* with both the *Abd-B* target gene and the *Fab-7*^*1*^deletion to observe the GOF phenotype. Surprisingly, the third class of revertants disrupted *iab-6* (Fig. 4e). This is the most interesting class regarding the *open for business* model, because it allowed for the ruling out of the simple hypothesis that the *Fab-7*^*1*^ deletion removes binding sites for a repressive complex negatively regulating *iab-7* in A6/PS11. If this had been the case, disruption of *iab-6* should not interfere with this simple de-repression mechanism. Furthermore, the normal appearance of *Abd-B* expression in A7/PS11 in this *iab-6* class of revertants clearly demonstrates that the *Fab-7*^*1*^ deletion does not affect essential sequences for proper *iab-7* activity. Based on this reversion experiment, it was concluded that in A6/PS11 of *Fab-7*^*1*^ flies, the *iab-6* and *iab-7* domains are fused into a single functional unit with mixed characteristics: parasegmental/segmental address is provided by *iab-6* and parasegmental/segmental identity is provided by *iab-7*. Revertants of *Fab-7*^*1*^ mapping further to the left in *iab-5* or *iab-4* were never recovered, indicating that *iab-6* worked in an autonomous fashion with respect to the *Fab-7*^*1*^ phenotype.

#### Additional boundary mutations

*Mcp*^*1*^ and *Fab-7*^*1*^ were discovered as spontaneous mutations probably because the identities of the affected abdominal segments are easily recognized in the adult fly. Although the open-for-business model predicts the existence of boundary elements flanking each of the nine regulatory domains, additional boundary mutations did not appear in traditional, non-directed, genetic screens. This is probably because the remaining abdominal segments look very similar, making homeotic transformations difficult to identify. Nevertheless, there are three additional boundaries in the abdominal region of the BX-C that are genetically characterized. The *Fab-8* boundary demarcates the *iab-7* from the *iab-8* regulatory domain (Barges et al. 2000). It was isolated by imprecise P-element excision using a P-element insertion line that was recovered on the basis of its sterility phenotype (Spradling et al. 1999). Fortuitously, this P-element inserted within *Fab-8*. Imprecise excision of this P-element showed that deletion of the region between *iab-7* and *iab-8* induces a partial transformation of A7 into A8 (Barges et al. 2000), again supporting the idea of a boundary element between *iab-7* and *iab-8*. The case of *Fab-6*, separating *iab-5* from *iab-6* is less straightforward. It was first functionally inferred on the DNA map by the differences in phenotype between two internal deficiencies sharing the same distal breakpoint (toward *Abd-B*) but differing at their proximal breakpoints (toward *abd-A*; (Mihaly et al. 2006)). Later, this region was cleanly deleted and flies mutant for *Fab-6* displayed a weak but consistent boundary phenotype (Iampietro et al. 2010). Finally, the *Fub* boundary marks the border between the *bxd/pbx* domain specifying A1 and the *iab-2* domain that specifies A2 (Bender and Lucas 2013). *Fub* mutations were recovered by targeted mutagenesis following a hypothesis-driven experiment (see below).

### Painting DNA domains of the BX-C with enhancer trap lines

From 1982 (Rubin and Spradling 1982) to 2006 (Groth et al. 2004), transgenesis in *Drosophila* was accomplished using P-element transposons as vectors. Insertion of the transgenic constructs was more or less random. Because of the promiscuous nature of enhancers and other chromatin regulatory elements (such as *Polycomb-*Response-Elements and heterochromatin), expression from the transgenes was often influenced by the neighboring chromosomal environment, a phenomenon known as position-effect (PE). The phenomenon of PE inspired Cahir O’Kane and Walter Gehring to engineer a lacZ-based reporter transposon aimed at trapping the activity of regulatory elements in the vicinity of the insertion site of a transposon (O’Kane and Gehring 1987). Using this P-element, O’Kane and Gehring, discovered that about 1/3 of the insertion lines gave rise to a lacZ expression pattern that was spatially and/or temporally restricted. This breakthrough observation opened up new avenues for identifying genes based on their expression pattern. Of the thousands of lines that have been generated in *Drosophila* laboratories across the world, only few landed in the BX-C.

The use of P-elements with lacZ reporter genes to study enhancers and other chromosomal regulatory elements led to the astonishing discovery of a phenomenon called homing, in which a DNA fragment can direct its insertion to the vicinity of the site from which it originates. Homing is rare and was first discovered with a fragment from the regulatory region of the *engrailed* gene (Hama et al. 1990; Kassis et al. 1992). Another such homing fragment is a 7-kb-long DNA fragment derived from the region between *bxd/pbx* and *iab-2*. In this case, 18 % of the P-element constructs carrying this homing fragment inserted into the BX-C (Bender and Hudson 2000). While the mechanisms behind homing remain elusive, it is worthwhile mentioning that the *homing pigeon* fragment spans the *Fub* boundary that separates the *bxd/pbx* regulatory domain from the *iab-2* domain (see above; Bender and Lucas 2013). The idea of boundaries mediating homing is further substantiated by a more recent case of homing discovered at the *eve* locus by Fujioka and Jaynes (Fujioka et al. 2009). In this case, the homing fragment spans the *homie boundary* that insulates the *eve* locus from the next adjacent gene TER94 (Fujioka et al. 2013).

**Fig. 5:**
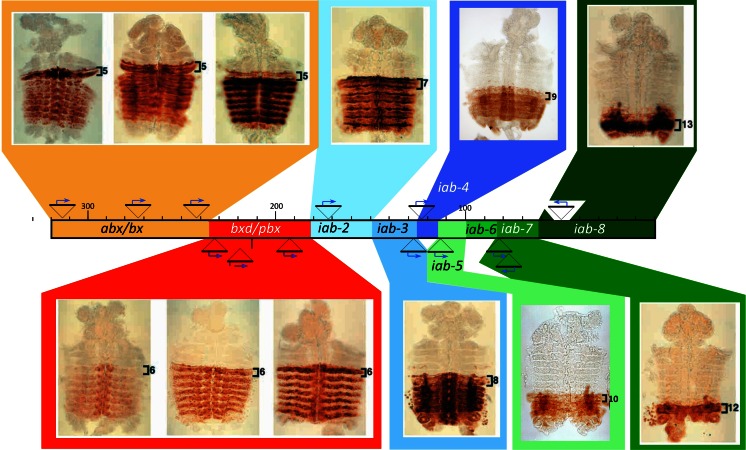
Painting the regulatory domains with enhancer trap lines. Reproduced from Fig. 5 of Maeda and Karch (2006); DOI: 10.1242/dev.02323 with the permission of the Company of Biologists. The 300-kb-long genomic DNA of the bithorax are represented as a *long rectangle* in the middle of the figures, with the insertion sites of the various enhancer trap P[lacZ] transposons indicated by *triangles* above it. Embryos stained with antibodies directed against ß-galactosidase are shown above and below the DNA lines. They were cut along the dorsal midlines and flattened on a microscope slide. The anterior parasegmental boundary of lacZ expression is indicated in each embryo. Note that this anterior border of expression moves by increment of 1 parasegment when the insertion site of the P[lacZ] transposon moves from left to right on the DNA map. The extent of each regulatory domain was determined by integrating the insertion sites of the P[lacZ] transposons with the locations of various rearrangement breakpoints associated with *iab* mutations and with the locations of the *Mcp*, *Fab-7*, *Fab-8* mutations

With the help of the *homing pigeon* fragment, the lab of Welcome Bender generated numerous new enhancers trap lines spread throughout the BX-C. Figure 5 shows some of these lines. The colored lines in this figure correspond to the DNA domains that are depicted in Fig. 2. If we focus on the three transposons inserted within the 75 kb region colored in orange, we find that the anterior border of expression of the lacZ reporter genes marks precisely PS5. This region comprises the sites of the *abx/bx* mutations that activate *Ubx* expression in PS5 and more posterior parasegments. Obviously, the promoters of the lacZ reporter genes in these three lines are trapping different sets of enhancers, as revealed by their different tissue specificities of expression. Nevertheless, all three enhancer trap lines share the same anterior border of expression in PS5. Meanwhile, the anterior border of expression of the next three enhancer trap lines (within the region colored in red) is shifted one parasegment posterior, in PS6. These three insertions map to the region previously assigned to the *bxd/pbx* region that controls *Ubx* expression in PS6. Once again, the tissue distribution and intensities of lacZ expression varies between the three lines but the anterior border of each starts at PS6.

By examining a large number of enhancer trap lines in the BX-C, Bender and Hudson (2000) made three major observations. First, enhancer trap lines that are spread over large distances often produce the same expression pattern, whereas other located just a few kb away produce a different pattern. This, for example, is the case for the rightmost transposon in the orange domain and the leftmost transposon in the red domain. These two transposons are located only a few kilobases apart but nevertheless express in different parasegments (PS5 and PS6 respectively; Fig. 5). Second, the anterior border of lacZ expression always progress toward the posterior by increment of one parasegment. And third, once an enhancer trap line is activated in a given parasegment, it remains active in the more posterior parasegments, following the first rule of Ed Lewis (see above). Taken together, the enhancer trap experiments provide additional visual evidence that there are distinct, and precisely definable domains of coordinated activity within the BX-C. As the known boundary elements mapped to the transition zones between domains, these experiments also helped to validate the idea that boundary elements limit the extent of domain activity.

### Additional boundaries in the BX-C

In Fig. 5, we took into account the positions of the enhancers trap insertion sites and the sites of mutations causing *iab* phenotypes to draw the extent of the regulatory domains. If, as mentioned above, boundary elements limit the extent of each domain, then we can infer the position of other boundary elements using this figure. P-elements in close proximity but expressed in different parasegments, give the most precise information for mapping boundaries. This is the case, for example, for the boundary separating the *abx/bx* domain (orange) from the *bxd/pbx* domain (red). This region contains the *Ubx* promoter. Similarly, the putative boundary (*Fab-*3) separating *iab-4* from *iab-5* can be localized accurately between the 2 transposons inserted on each side of it and that are expressed in PS9 and PS10, respectively.

**Fig. 6:**
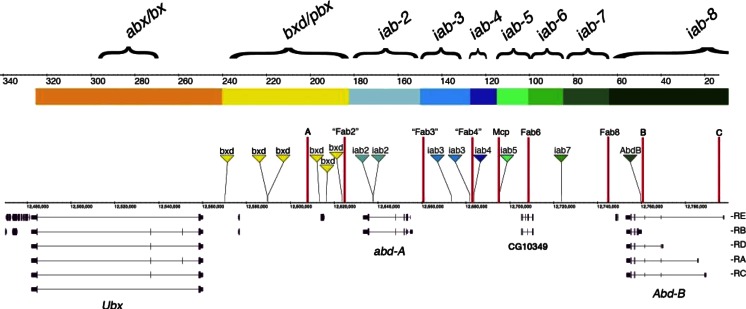
Position of CTCF sites in the BX-C as determined by chip’n chips. Picture taken from Holohan et al. (2007); DOI: 10.1371/journal.pgen.0030112 and reproduced with the permission of PLoS Genetics. The representation of the BX-C genomic region is taken from our review article published in 2006 in Development (Maeda and Karch 2006). The corresponding genomic region as described in Flybase (http://flybase.org/) is reproduced below the painted BX-C genomic region. Note the presence of CTCF binding in all the boundaries to the exception of *Fab-7* (see text). It should be noticed that boundary deletions of *Fab-2* have been since then recovered and named *Fub* (Bender and Lucas 2013) in reference to the *Ultra-abdominal (Uab)* alleles initially identified by Lewis (1978)

In 2007, the laboratory of Rob White performed a whole genome search for chromatin sites associated with the CTCF insulator factor (Holohan et al. 2007). As BX-C boundaries have been shown to behave as insulators in ectopic contexts, the White lab spent some part of their analysis on the distribution of CTCF sites within the BX-C. Using a figure based on Hudson and Bender’s mapping data, they described an almost perfect match between the boundaries as shown in Fig. 5 and the presence of CTCF sites. It appears then that *Fub*, *Fab-2*, *Fab-3*, *Fab-4*, *Mcp*, and *Fab-8* are all highlighted by the presence of CTCF binding sites (Fig. 6). Surprisingly, the best characterized boundary *Fab-7*, represents a conspicuous exception to this rule.

### Initiator elements function as “domain control regions”

Using transgenic approaches with lacZ reporter genes, several laboratories searched the BX-C regulatory regions for new and important regulatory elements. Among the elements identified were early embryonic enhancers (initiators), cell-type-specific enhancers, silencers and insulators (Simon et al. 1990) (Muller and Bienz 1992) (Busturia and Bienz 1993) (Zhou et al. 1996) (Hagstrom et al. 1996) (Fritsch et al. 1999) (Zhou et al. 1999) (Barges et al. 2000) (Horard et al. 2000) (Shimell et al. 2000) (Gruzdeva et al. 2005) (Mihaly et al. 2006). What was surprising from these analyses was that there were very few elements discovered that were restricted along the A-P axis. For example, an individual cell-type-specific enhancer might drive expression only in neuroblasts, but this expression was not restricted along the A-P axis. Likewise, the silencers and insulators discovered would perform their activity irrespective of A-P position. In fact, when any domain was dissected, one could expect to find only one or two elements within each domain that were limited along the A-P axis. These rare elements, now called initiators, had the ability to turn on reporter gene expression from the specific parasegment controlled by the *iab* domain from which it was isolated, and in more posterior parasegments (Busturia and Bienz 1993; Mihaly et al. 2006; Simon et al. 1990). From the transgenic analysis alone, a mystery developed on how a group of non-AP restricted enhancers could be used to determine an AP restricted event (i.e., the creation of a specific segment). However, the transgenic analysis fit perfectly with predictions of the *open for business* model. According to the *open for business* model, most BX-C enhancers should be naïve to AP position, relying instead upon some spatial cue to come from the domain in which it resides. The only thing missing from this model was the identity of that cue and how the whole domain perceived its AP position. As the only elements found in BX-C that autonomously respond to an AP position, the early embryonic enhancer/initiators were proposed to read a parasegmental address and to communicate this knowledge to the rest of the elements within a domain.

**Fig. 7:**
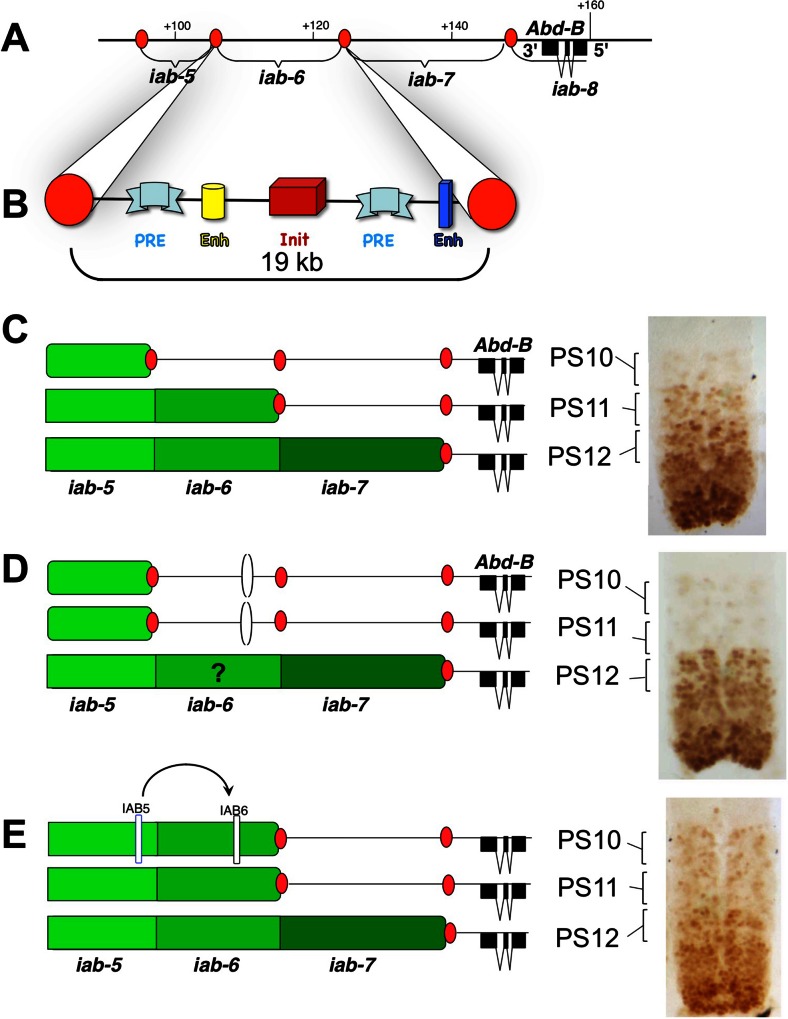
Initiators function as domain control regions. Figure reproduced from the review article of Maeda and Karch (2011); DOI: 10.1016/j.gde.2011.01.021, with the permission of Elsevier. This article was reviewing work published by our laboratory in 2010 (Iampietro et al. 2010). The *Abd-B* genes and associated regulatory regions *iab-5*, *iab-6*, *iab-7*, and *iab-8* regulatory regions is drawn in panel **a** with a central nervous system (CNS) dissected out of an embryo stained with antibodies against *Abd-B* (see also legend of Fig. 4 for the parasegmental expression of *Abd-B* in the CNS.) In panel **b**, a magnification of the 19-kb-long *iab-6* domain is drawn in the form of a cartoon. *Ovals* indicate boundaries. PREs, cell type-specific enhancer (Enh) and initiator elements (Init) are drawn. Panel **c** shows the sequential opening of the *iab-5*, *iab-6*, and *iab-7* regulatory domains in PS10, PS11, and PS12, respectively. Panel **d** shows the consequence of deleting the *iab-6* initator alone (a 927-bp-long deletion). Despite the fact that 18.1 kb of *iab-6* remains intact, the whole domain seems inactive as revealed by the *Abd-B* expression pattern in PS11 which is a reiteration of the expression observed in PS10. In agreement with this embryonic phenotype, the adult flies emerge with a complete transformation of A6 into A5. The initiator swapping experiment is shown in panel **e**. In this strain, the *iab-6* initiator was removed and replaced by the initiator of *iab-5*. Note the PS10 *Abd-B* expression pattern that is similar to the pattern normally present in PS11, indicating that the *iab-6* domain is opened in PS10. In agreement with this effect in embryos, adult flies emerge with a transformation of A5 into A6. In these initiator swapping flies, the parasegmental address is provided by the *iab-5* initiator and the segmental identity is provided by the cell-type-specific enhancers of *iab-6*

If initiator elements truly perform this function, then there are certain predictions that can be made. First, the removal of an initiator from a domain should completely abolish the activity of the whole domain. And second, switching an initiator from one domain for the initiator of a more anterior domain should cause activation of the more posterior domain in the parasegment specified by the more anterior initiator. We have directly addressed both of these predictions using a technique that coupled homologous recombination and ΦC31 site-specific integration to target the *iab-6* regulatory domain for mutagenesis (Iampietro et al. 2010). Using this method, we showed that removal of the *iab-6* initiator (a 927 bp fragment) abolishes *iab-6* function, even though 18 kb of *iab-6* sequences remains. This results in an A6 to A5 transformation, as the *iab-5* domain is active in parasegments posterior to A5/PS10 (Fig. 7d). Next, we showed that switching the *iab-6* initiator with that of *iab-5* caused the enhancers present in *iab-6* to become active one parasegment too anterior, in PS10(A5). This caused a A5 to A6 transformation, as seen in Fig. 7e. Thus, our study proved that initiators function as a “domain control regions” to read A-P positional information and accordingly, coordinate the various enhancers (which pattern the parasegment) within a domain. How initiators accomplish this feat remains to be discovered, but clearly, our data suggests a hierarchical nature to the regulatory elements in the BX-C consistent with the predictions of the *open for business* model.

### H3K27 modifications define segmental regulatory domains in the *Drosophila* bithorax complex

Since its conception, the open-for-business model always proposed that enhancer domains somehow *open* in parasegments where they should be active. Thus far, we have described the evidence supporting a parasegment-specific activation of chromosomal domains and how these domains sense a parasegmental address. What we have not described is what it means to be active or *open*. To do this, we must first introduce the last group of players in this puzzle: the *Polycomb-Group* (*Pc-G*) and *trithorax-Group* (*trx-G*) genes.

During embryogenesis, the bithorax complex is thought to go through two phases of regulation: an early, initiation phase and a later, maintenance phase (see Maeda and Karch 2006 for review). Due to the timing of bithorax gene expression, the initiation phase is thought to be under the control of the transcription factors encoded by the maternal, gap and pair-rule genes that are responsible for the subdivision of the early embryos into 14 parasegments (for reviews, see for example Ingham, 1988; Hoch and Jackle, 1993; Kornberg and Tabata, 1993; DiNardo et al., 1994). These transcription factors are thought to interact with the initiators of each *cis*-regulatory domain to determine their ultimate expression pattern along the AP axis (Casares and Sanchez-Herrero 1995; Irish et al. 1989; Shimell et al. 1994; White and Lehmann 1986). For example, the combination of gap and pair-rule gene products present in PS11 are thought to bind to the *iab-6* initiator to allow the *iab-6 cis*-regulatory region to control *Abd-B* expression in PS11/A6, while at the same time preventing the *iab-7 cis*-regulatory region from becoming active. Supporting this view, initiator elements do seem to contain numerous binding sites for these early transcription factors and in a few cases, have been shown to be dependent upon the activity of these transcription factors (Busturia and Bienz 1993; Ho et al. 2009; Qian et al. 1991; Shimell et al. 1994; Starr et al. 2011).

However, because the gap and pair-rule genes are only transiently expressed in the early embryo, and the activity states of the segment-specific *cis*-regulatory regions seem to be fixed for the life of the fly, a system to maintain homeotic gene expression is required within each *cis*-regulatory domain (Struhl and Akam 1985). The maintenance of homeotic gene expression has been shown to require the products of the *Pc-G* and *trx-G* genes. While the *Pc-G* products are thought to function as negative regulators, maintaining the inactive state of the *cis*-regulatory regions not in use, the *trx-G* products function as positive regulators, maintaining the active state of active regulatory regions (Kennison 1993; Paro 1990; Pirrotta 1997; Simon 1995). Both the *Pc-G* and *trx-G* products are known to bind within the parasegment-specific *cis*-regulatory domains to specific elements called *Polycomb*/*Trithorax* Response Elements (PREs/TREs) and are thought to maintain the active or inactive state of each domain by modifying its chromatin structure (Brown and Kassis 2013; Muller and Kassis 2006; Schwartz and Pirrotta 2008; Simon and Kingston 2009).

*Pc-G* proteins have been shown to form distinct chromatin repressive complexes (PRC1 and PRC2) with distinct chromatin modifying activities. While the PRC1 complex seems to ubiquitylate histone H2A (de Napoles et al. 2004), (Scheuermann et al. 2010) PRC2 seems to primarily methylate histone H3 on lysine K27 (Czermin et al. 2002; Muller et al. 2002; Ng et al. 2000). Although the molecular details on how these epigenetic changes may result in chromatin compaction and lowering gene expression remain poorly understood, it is known that both of these chromatin marks correlate with repressive chromatin environments.

Association of the *Polycomb* protein with the chromatin of the BX-C was first shown in 1993 by chromatin immuno-precipitations (ChIP) experiments performed with *Drosophila* tissue culture cells (Orlando and Paro 1993). Similar ChIP experiments performed in *Drosophila* Kc cells showed that the mark of PRC2, H3K27me3, was also present in the BX-C. Interestingly, this chromatin mark covered all of the BX-C, suggesting that in Kc cells, the whole BX-C was repressed. Later, when ChIP data was analyzed from a different *Drosophila* cell line, the SF4 cells, only the *Ubx* and *abd-A* portion of the BX-C was covered by H3K27me3 marks. The *Abd-B* gene and its associated *iab-5* through *iab-8* regulatory domains was completely devoid of H3K27me3, and conversely was associated with hyperacetylation of histone H4 (H4Ac), a mark associated with active genes (Beisel et al. 2007; Schwartz et al. 2006). This epigenetic signature fit well with the expression profil of the BX-C Hox genes in these two cell lines. While Kc cells do not express any BX-C homeotic genes, Sf4 cells express exclusively *Abd-B*. Later work, comparing tissue from the wing and haltere discs also supported a correlation between BX-C homeotic gene expression, in this case *Ubx*, and the lack of the H3K27me3 mark (Papp and Muller 2006). However, there was one problem. Based on the *open for business* model, one would expect that the H3K27me3 marks should be progressively stripped off from the chromatin by an increment of one domain at a time as one moves from anterior to posterior along the AP axis. The work from the cell lines seemed to slightly contradict this prediction, as only the *Abd-B* region seemed to lack H3K27me3 and show H4Ac. The work in the discs did not resolve this discrepancy as they only examined the *Ubx* region of the BX-C in anterior tissues where no other BX-C gene should be *open*. Therefore, a real test for domain opening of the *open for business* was still needed. Unfortunately, to truly test the *open for business* model, experiments would have to be done using purified populations of cells derived from different parasegments of the embryo spanning domains of expression of more than one BX-C homeotic gene. Until recently, this task seemed impossible due to the difficulty of separating and sorting *Drosophila* embryonic cells.

In 2010, Deal and Henikoff developed a system called INTACT to bypass this problem for ChIP by sorting specific populations of *Drosophila* embryonic nuclei. This method uses a nuclear envelope protein expressed under the control of a cell-type-specific promoter to anchor an mCherry marker with the biotin ligase recognition peptide (BLRP). After tissue disruption, specific nuclei are sorted by FACS or by affinity purification on streptavidin columns (Deal and Henikoff 2010). While in *Drosophila*, the vast number of Gal4 driver lines allows the INTACT marker to be expressed in almost any cell-type or tissue, it is often a problem to limit this expression to exclude cells that are not of interest. This was the challenge in the BX-C: to express the marker in all of the various cell-tyes in a parasegment, but to exclude the cells of neighboring parasegments. Lessons from the lacZ enhancer trap lines showed that transgenes inserted into the BX-C could drive expression of a marker in a particular parasegment. However, as the enhancer trap lines also revealed, these drivers would remain active in all the posterior parasegments (as predicted by Ed Lewis’ first rules).

**Fig. 8:**
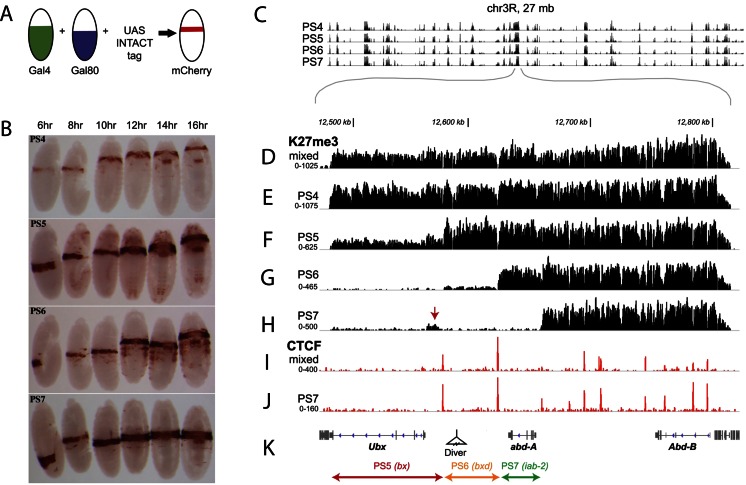
H3K27 modifications define segment-specific regulatory domains. The figure compiles Figs. 2 and 3 of Bowman et al. (2014); DOI 10.7554/eLife.02833 reproduced with the permission of eLife. Panel **a** shows the strategy to obtain strains expressing active yeast Gal4 activator in single parasegments. The homing fragment was used to attract transposons harboring either the Gal4 activator or the Gal80 repressor in the regulatory domains (see detailed procedure in figure supplement 1 of Bowman et al. 2014). Drivers for the Gal4 activator or the Gal80 repressors each with different anterior limit of expressions are combined by simple crosses (see also text). Panel **b** shows the resulting expression pattern for strains expressing active Gal4 in PS4, PS5, PS6, and PS7, respectively. Note the existence of weak leakiness in anterior parasegments in the PS5-specific combination (see remark below). These Gal4 strains active in single parasegments are then crossed to INTACT construct (Deal and Henikoff 2010) to purify nuclei from single parasegment and perform ChIP-seq experiments with antibodies recognizing H3K27me3 modification. Panel **c** is a control experiment revealing that the overall H3K27me3 profile over a region of 27 Mb centered around the BX-C is invariant in the nuclei isolated from PS4, PS5, PS6, and PS7. Panels **d** through **h** show the H3K27me3 profile over the entire BX-C. The H3K27me3 profile from whole embryo (panel **d**) does not differ from the profile obtained from PS4 nuclei (panel **e**) where the entire BX-C is repressed. In PS5 however (panel **f**), the H3K27me3 profile is greatly reduced over the PS5-specific regulatory domain (as indicated in **k**). The fact that the H3K27me3 profile does not reach the background levels seen in the more posterior domains (panel **g** and **h**) probably stems from the leakiness of the PS5 specific driver in anterior parasegments, suggesting that the preparation is contaminated with nuclei originating from anterior inactive regions. Note the progressive loss of H3K27me3 modifications in nuclei derived from PS6 and PS7 (panel **g** and **h**, respectively). Panel**s i** and **j** show that the CTCF binding profiles do not differ in nuclei isolated from PS7 and mixed nuclei isolated from whole embryos, suggesting that this boundary factor is bound in a constitutive fashion, regardless of the state of activity of the regulatory domans of the BX-C

Through the work of the Bender and Kingston lab, this problem was finally solved (Bowman et al. 2014). As mentioned above, reporters inserted into the BX-C express according to the activity of the domain in which they are inserted. As such, the reporter starts its expression in one parasegment and continues throughout all of the parasegments more posterior. Thus, if a Gal4 reporter was inserted into the *abx/bx* domain, it would express the Gal4 activator from PS5 until the posterior end of the embryo. Meanwhile, a Gal4 inserted into the *bxd/pbx* domain would express from PS6 until the posterior end of the embryo. What Bender’s group did, was to create double insert lines where a Gal4 transgene was inserted into one *cis*-regulatory domain and a Gal80 expressing transgene (an inhibitor of Gal4 activity) was inserted into the next more posterior domain (Fig. 8a). In this way, they were able to express Gal4 in a broad region of the embryo, but have it only active in one parasegment (Bowman et al. 2014) (Fig.8b). Of course, this was much harder to do than it seems on paper, as transgenes inserted into the BX-C often trap different tissue-specific enhancers, depending upon where in the domain the transgenes inserted. Still, through the tenacious fine tuning of the Bender group, a set of lines having Gal4 activity exclusively in PS4, PS5, PS6 or PS7 was finally created.

Using these lines, nuclei were isolated from individual parasegments for ChIP-seq experiments. H3K27me3 ChIP on these samples confirmed the remarkable domain *opening*, predicted by the domain model (Fig. 8c through h). Nuclei derived from PS4 had the whole BX-C covered with H3K27me3 (Fig. 8e). Nuclei derived from PS5 had H3K27me3 retracting from the area of the chromosome attributed to the PS5 controlling *abx/bx* domain (Fig. 8f). Nuclei derived from PS6 had H3K27me3 retracting from the area attributed to both the *abx/bx* domain and the *bxd/pbx* domain (controlling *Ubx* in PS6; Fig. 8g). And finally, nuclei derived from PS7 had H3K27me3 retracting from the area from spanning the *abx/bx* domain until the *iab-2* domain (Fig. 8h) (Bowman et al. 2014).

With regards to the *open for business* model, the work of Bowman et al. confirmed a number of important details. First was the precision in which a domain was activated. In their experiment, Bowman et al. found that the retracting H3K27me3 signal essential went from one point on the chromosome to another, without any sloping intermediate zones. ChIP experiments directed against the boundary protein CTCF confirmed that these places of abrupt transition coincided with expected boundary elements. Next, in the absence of H3K27me3, H3K27 acetylation marks were found. As H3K27Ac is a mark associated with active chromatin, it seems like domains that are not silenced become active, or *open*. Lastly, these experiments showed that even in parasegments where a given homeotic gene is not the primary segment-determining gene, domains controlling more anterior homeotic genes are still active. This was seen in the PS7 ChIP experiments where the *abx/bx* and *bxd/pbx* domains remained active, even though it is *iab-2* controlling *abd-A* expression that is the primary determinant of PS7 identity.

### “Sure enough, I was [we were] right.”

To spatially regulate the activation of homeotic genes along the AP axis, the open for business model proposes that enhancer containing domains open sequentially along the chromosome as one follows the anterior-posterior axis of the fly (Fig. 2). At first glance, this model seems quite simple to comprehend. Yet, within this model lie numerous implications, implications that have taken over 25 years to validate. Through the experiments that have been described in this review, we can now see many of the hidden details inherent in the open for business model. First, each domain contains a core set of regulatory elements that function in a hierarchical manner. At the bottom of this hierarchy seems to be the cell-type-specific enhancers that turn on single homeotic genes in cells appropriate for a specific parasegment. Controlling these enhancers are the PRE silencers and TREs that either compact the enhancers into a heterochromatin-like structure in parasegments where they are not needed or open the domain in parasegments where they should be active. To keep the domains separate are domain boundary elements that prevent the spreading of active or inactive chromatin from one domain to another. And lastly, there are the initiators that somehow instruct the PREs/TREs where they should or should not be active.

Although many questions still remain about the mechanisms by which each of the elements perform their function, the open for business model has been, for the most part, validated. As part of the community of researchers who contributed to the creation and validation of this model, this has been quite comforting. In our lab, we have a term for the experience of making a startling prediction that proves to be true. We call such instances, “Walter Gehring moments”, in honor of the numerous instances where Walter Gehring and his colleagues made incredible predictions that, through the elegant fusion of genetics and molecular biology, were proven to be true. For this reason, it seems quite fitting that this review to be placed in a series of articles dedicated to his memory.
